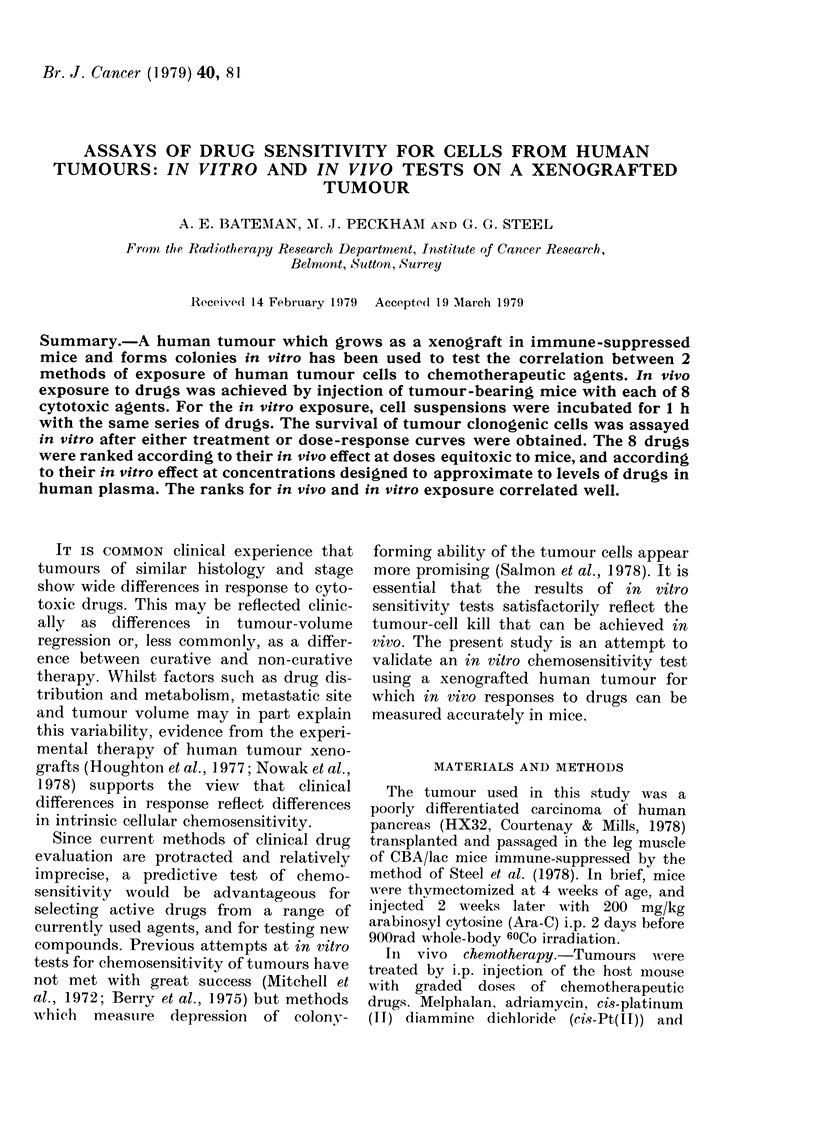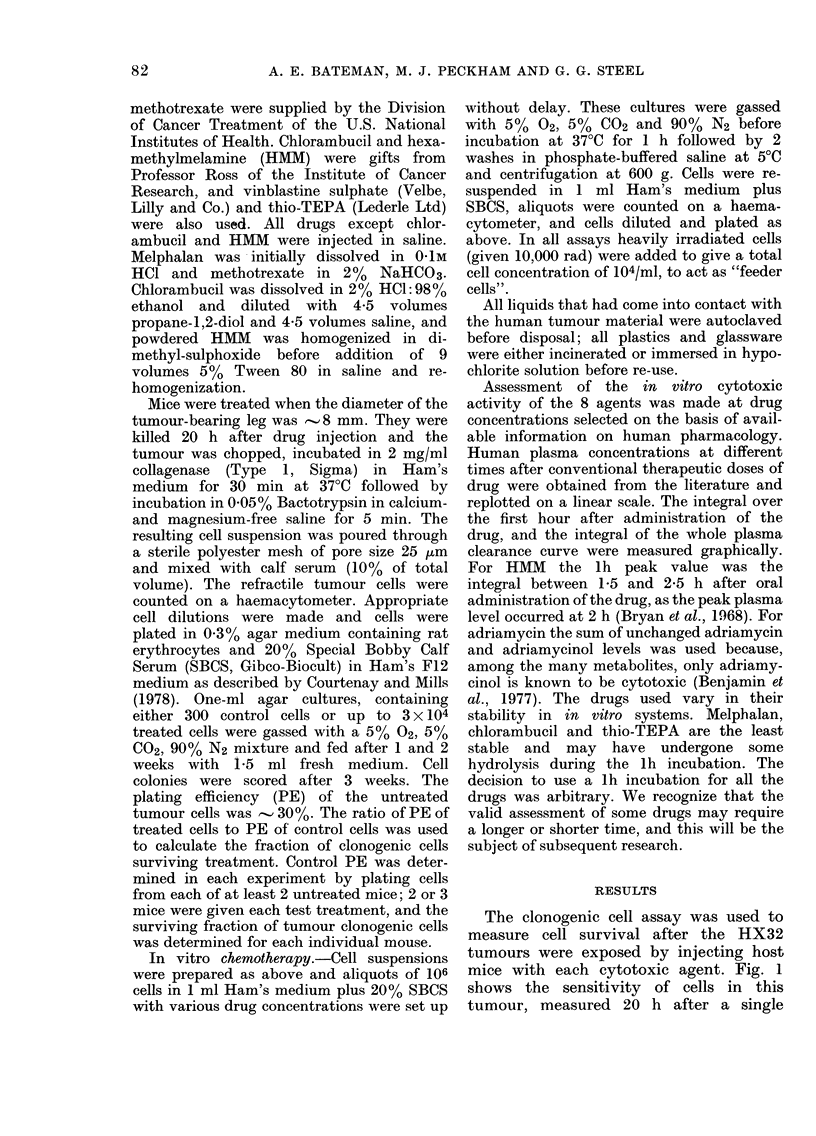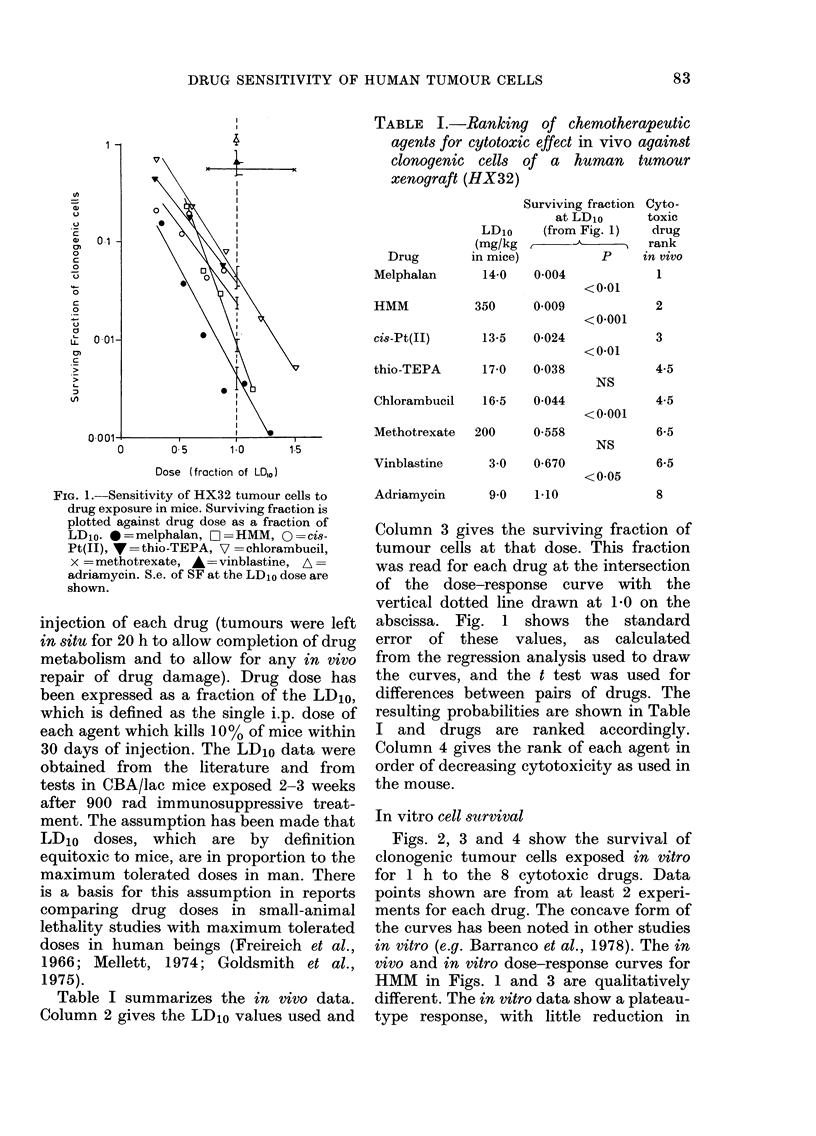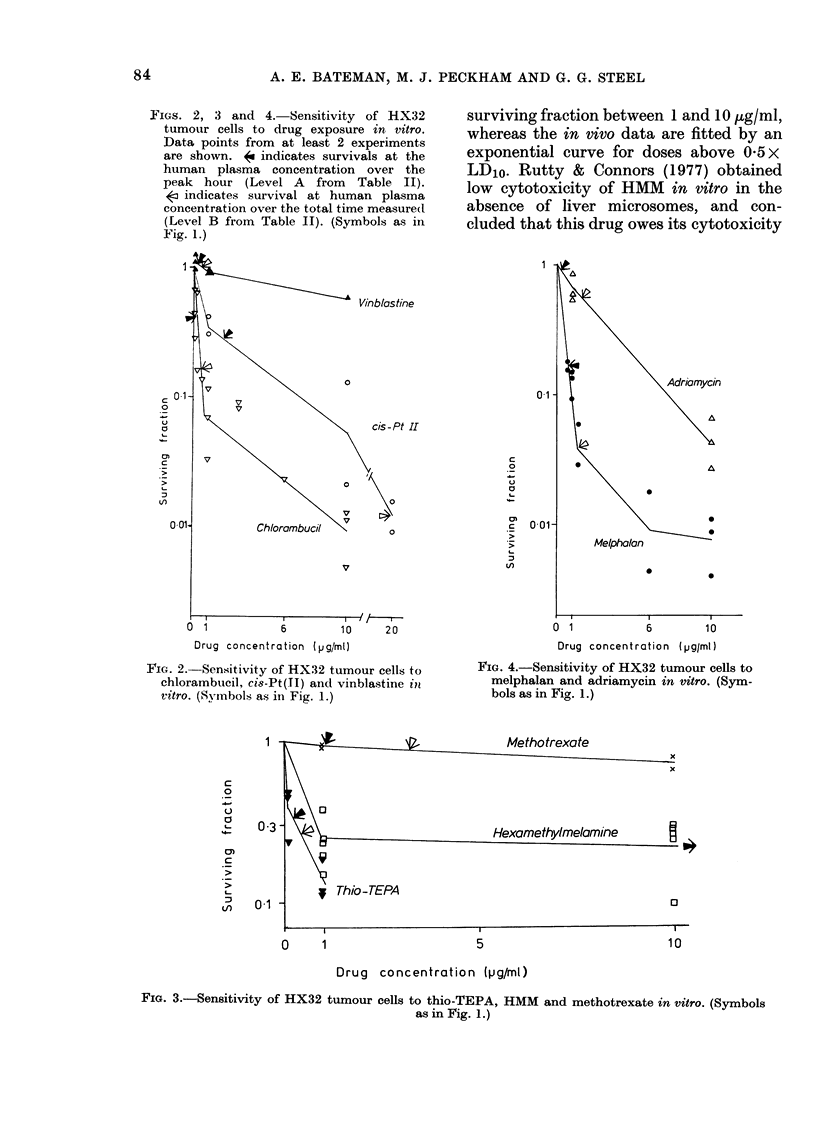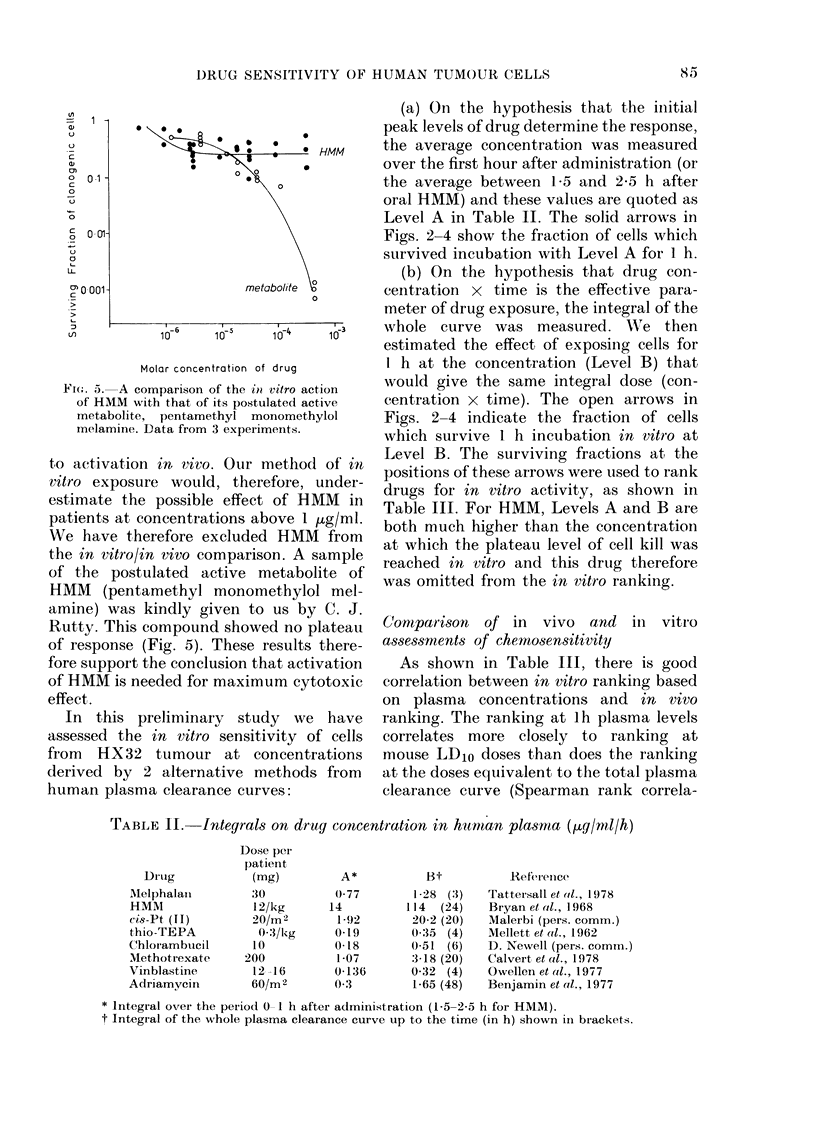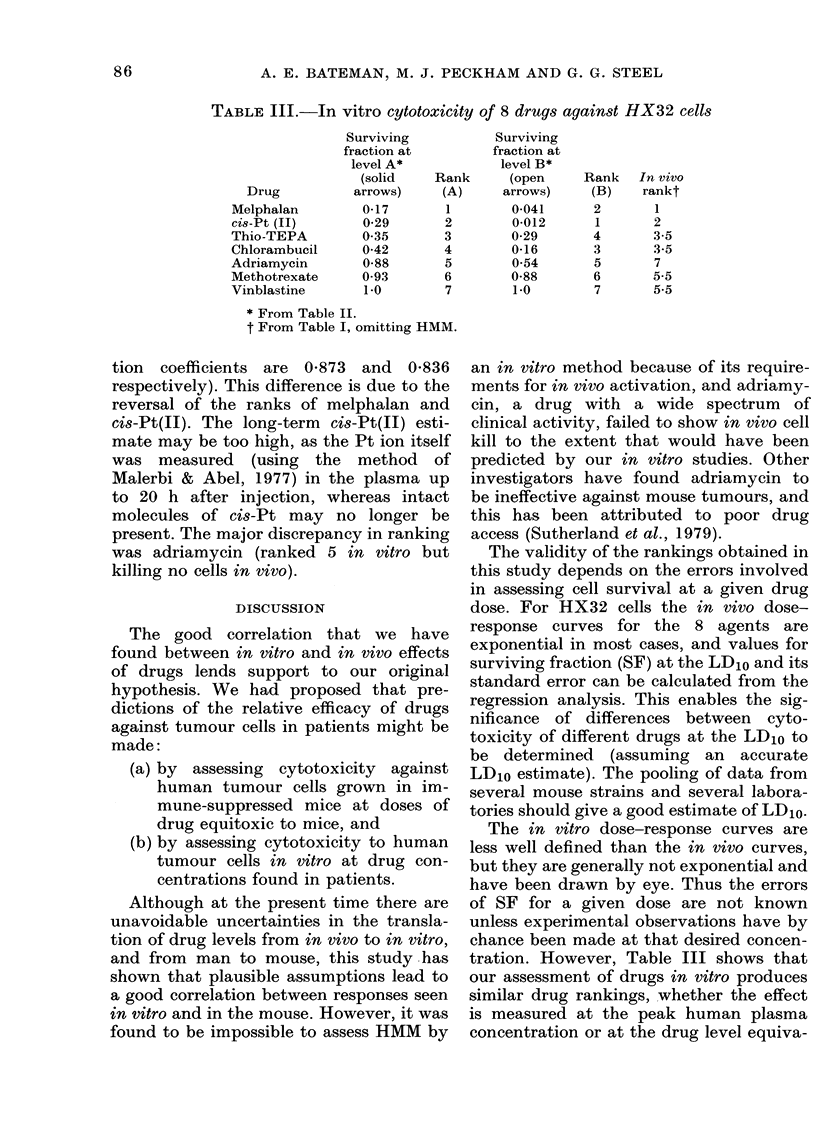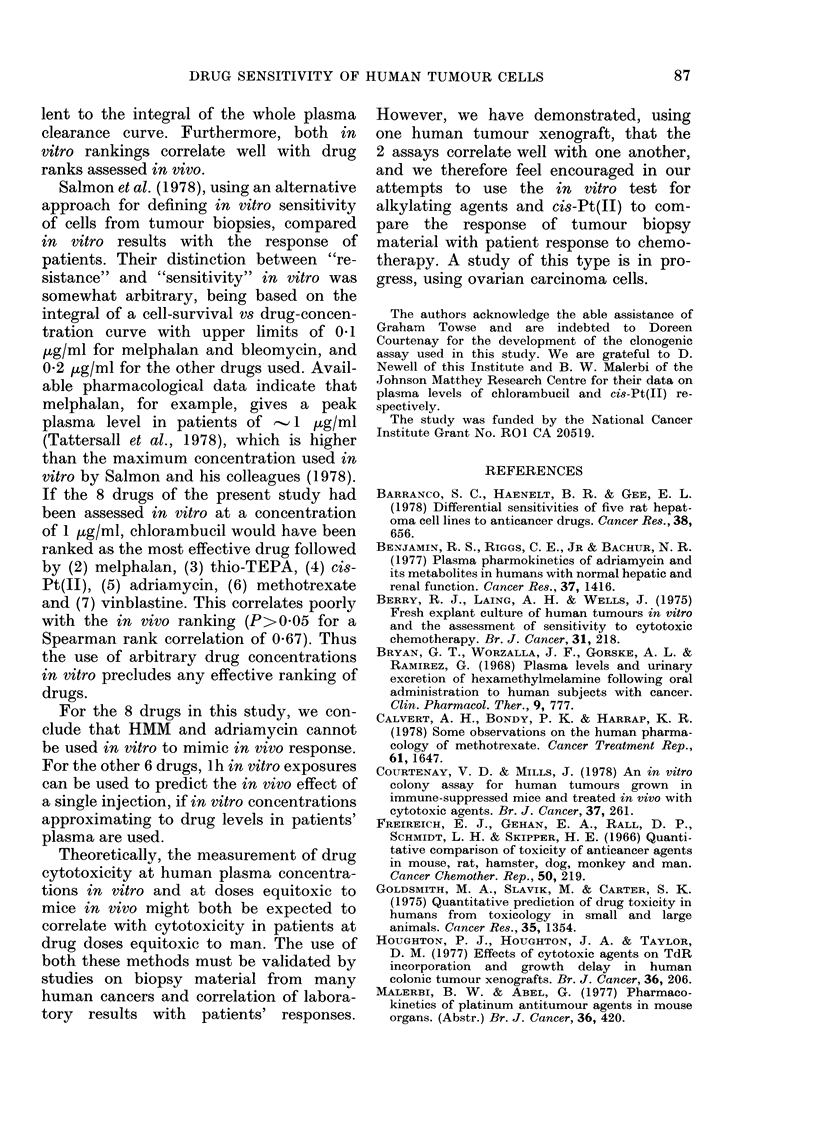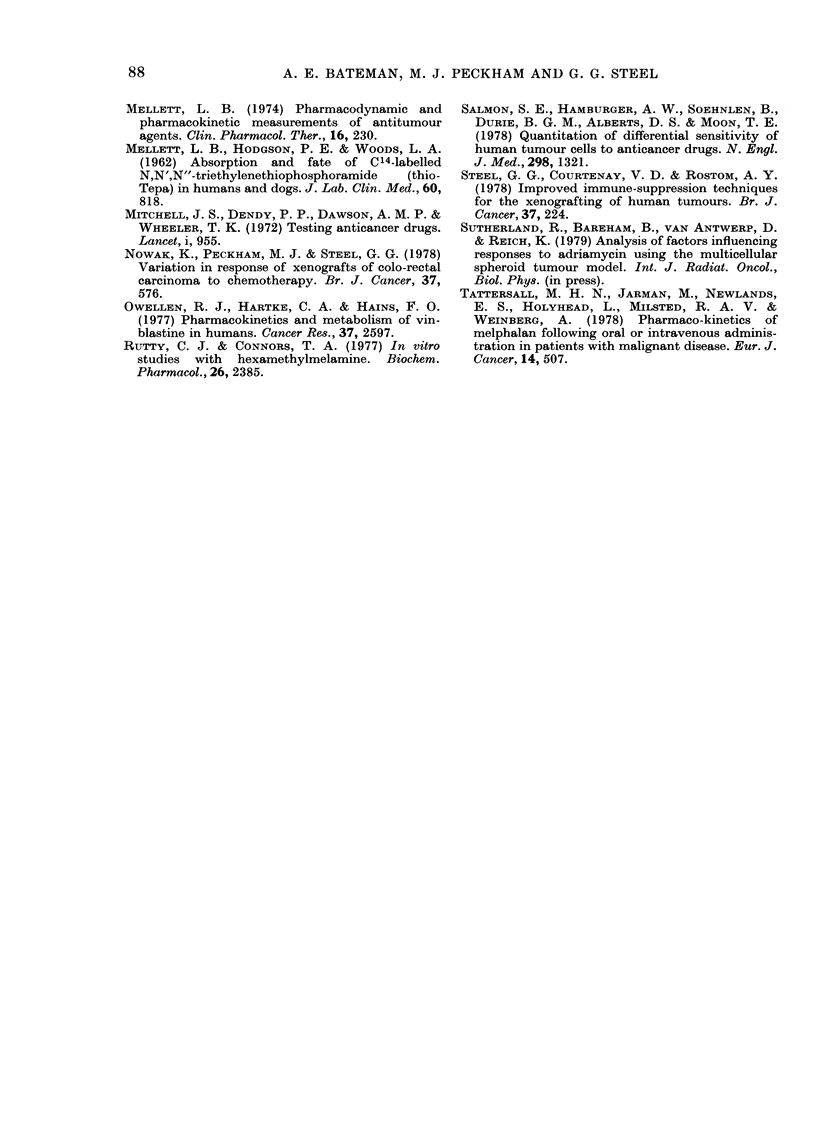# Assays of drug sensitivity for cells from human tumours: in vitro and in vivo tests on a xenografted tumour.

**DOI:** 10.1038/bjc.1979.143

**Published:** 1979-07

**Authors:** A. E. Bateman, M. J. Peckham, G. G. Steel

## Abstract

A human tumour which grows as a xenograft in immune-suppressed mice and forms colonies in vitro has been used to test the correlation between 2 methods of exposure of human tumour cells to chemotherapeutic agents. In vivo exposure to drugs was achieved by injection of tumour-bearing mice with each of 8 cytotoxic agents. For the in vitro exposure, cell suspensions were incubated for 1 h with the same series of drugs. The survival of tumour clonogenic cells was assayed in vitro after either treatment or dose-response curves were obtained. The 8 drugs were ranked according to their in vivo effect at doses equitoxic to mice, and according to their in vitro effect at concentrations designed to approximate to levels of drugs in human plasma. The ranks for in vivo and in vitro exposure correlated well.


*Br. J. Cancer* (1979) **40,** 81

### ASSAYS OF DRUG SENSITIVITY FOR CELLS FROM HUMAN TUMOURS: *IN VITRO* AND *IN VIVO* TESTS ON A XENOGRAFTED TUMOUR

A. E. BATEMAN, M. J. PECKHAM AND G. G. STEEL

*From the Radiotherapy Research Department, Institute of Cancer Research, Belmont, Sutton, Surrey*

Received 14 February 1979 Accepted 19 March 1979

**Summary.—A human tumour which grows as a xenograft in immune-suppressed mice and forms colonies *in vitro* has been used to test the correlation between 2 methods of exposure of human tumour cells to chemotherapeutic agents. *In vivo* exposure to drugs was achieved by injection of tumour-bearing mice with each of 8 cytotoxic agents. For the *in vitro* exposure, cell suspensions were incubated for 1 h with the same series of drugs. The survival of tumour clonogenic cells was assayed *in vitro* after either treatment or dose-response curves were obtained. The 8 drugs were ranked according to their *in vivo* effect at doses equitoxic to mice, and according to their *in vitro* effect at concentrations designed to approximate to levels of drugs in human plasma. The ranks for *in vivo* and *in vitro* exposure correlated well.**

It is common clinical experience that tumours of similar histology and stage show wide differences in response to cytotoxic drugs. This may be reflected clinically as differences in tumour-volume regression or, less commonly, as a difference between curative and non-curative therapy. Whilst factors such as drug distribution and metabolism, metastatic site and tumour volume may in part explain this variability, evidence from the experimental therapy of human tumour xenografts (Houghton *et al.*, 1977; Nowak *et al.*, 1978) supports the view that clinical differences in response reflect differences in intrinsic cellular chemosensitivity.

Since current methods of clinical drug evaluation are protracted and relatively imprecise, a predictive test of chemosensitivity would be advantageous for selecting active drugs from a range of currently used agents, and for testing new compounds. Previous attempts at *in vitro* tests for chemosensitivity of tumours have not met with great success (Mitchell *et al.*, 1972; Berry *et al.*, 1975) but methods which measure depression of colony-forming ability of the tumour cells appear more promising (Salmon *et al.*, 1978). It is essential that the results of *in vitro* sensitivity tests satisfactorily reflect the tumour-cell kill that can be achieved *in vivo.* The present study is an attempt to validate an *in vitro* chemosensitivity test using a xenografted human tumour for which *in vivo* responses to drugs can be measured accurately in mice.

### MATERIALS AND METHODS

The tumour used in this study was a poorly differentiated carcinoma of human pancreas (HX32, Courtenay & Mills, 1978) transplanted and passaged in the leg muscle of CBA/lac mice immune-suppressed by the method of Steel *et al.* (1978). In brief, mice were thymectomized at 4 weeks of age, and injected 2 weeks later with 200 mg/kg arabinosyl cytosine (Ara-C) i.p. 2 days before 900rad whole-body $^{60}$Co irradiation.

In vivo *chemotherapy.*—Tumours were treated by i.p. injection of the host mouse with graded doses of chemotherapeutic drugs. Melphalan, adriamycin, *cis*-platinum (II) diammine dichloride (*cis*-Pt(II)) and methotrexate were supplied by the Division of Cancer Treatment of the U.S. National Institutes of Health. Chlorambucil and hexamethylmelamine (HMM) were gifts from Professor Ross of the Institute of Cancer Research, and vinblastine sulphate (Velbe, Lilly and Co.) and thio-TEPA (Lederle Ltd) were also used. All drugs except chlorambucil and HMM were injected in saline. Melphalan was initially dissolved in 0·1M HCl and methotrexate in 2% $NaHCO_3$. Chlorambucil was dissolved in 2% HCl:98% ethanol and diluted with 4·5 volumes propane-1,2-diol and 4·5 volumes saline, and powdered HMM was homogenized in dimethyl-sulphoxide before addition of 9 volumes 5% Tween 80 in saline and rehomogenization.

Mice were treated when the diameter of the tumour-bearing leg was ~8 mm. They were killed 20 h after drug injection and the tumour was chopped, incubated in 2 mg/ml collagenase (Type 1, Sigma) in Ham's medium for 30 min at 37°C followed by incubation in 0·05% Bactotrypsin in calcium- and magnesium-free saline for 5 min. The resulting cell suspension was poured through a sterile polyester mesh of pore size 25 $\mu$m and mixed with calf serum (10% of total volume). The refractile tumour cells were counted on a haemacytometer. Appropriate cell dilutions were made and cells were plated in 0·3% agar medium containing rat erythrocytes and 20% Special Bobby Calf Serum (SBCS, Gibco-Biocult) in Ham's F12 medium as described by Courtenay and Mills (1978). One-ml agar cultures, containing either 300 control cells or up to $3\times10^4$ treated cells were gassed with a 5% $O_2$, 5% $CO_2$, 90% $N_2$ mixture and fed after 1 and 2 weeks with 1·5 ml fresh medium. Cell colonies were scored after 3 weeks. The plating efficiency (PE) of the untreated tumour cells was ~30%. The ratio of PE of treated cells to PE of control cells was used to calculate the fraction of clonogenic cells surviving treatment. Control PE was determined in each experiment by plating cells from each of at least 2 untreated mice; 2 or 3 mice were given each test treatment, and the surviving fraction of tumour clonogenic cells was determined for each individual mouse.

In vitro *chemotherapy.*—Cell suspensions were prepared as above and aliquots of $10^6$ cells in 1 ml Ham's medium plus 20% SBCS with various drug concentrations were set up without delay. These cultures were gassed with 5% $O_2$, 5% $CO_2$ and 90% $N_2$ before incubation at 37°C for 1 h followed by 2 washes in phosphate-buffered saline at 5°C and centrifugation at 600 g. Cells were resuspended in 1 ml Ham's medium plus SBCS, aliquots were counted on a haemacytometer, and cells diluted and plated as above. In all assays heavily irradiated cells (given 10,000 rad) were added to give a total cell concentration of $10^4$/ml, to act as "feeder cells".

All liquids that had come into contact with the human tumour material were autoclaved before disposal; all plastics and glassware were either incinerated or immersed in hypochlorite solution before re-use.

Assessment of the *in vitro* cytotoxic activity of the 8 agents was made at drug concentrations selected on the basis of available information on human pharmacology. Human plasma concentrations at different times after conventional therapeutic doses of drug were obtained from the literature and replotted on a linear scale. The integral over the first hour after administration of the drug, and the integral of the whole plasma clearance curve were measured graphically. For HMM the 1h peak value was the integral between 1·5 and 2·5 h after oral administration of the drug, as the peak plasma level occurred at 2 h (Bryan *et al.*, 1968). For adriamycin the sum of unchanged adriamycin and adriamycinol levels was used because, among the many metabolites, only adriamycinol is known to be cytotoxic (Benjamin *et al.*, 1977). The drugs used vary in their stability in *in vitro* systems. Melphalan, chlorambucil and thio-TEPA are the least stable and may have undergone some hydrolysis during the 1h incubation. The decision to use a 1h incubation for all the drugs was arbitrary. We recognize that the valid assessment of some drugs may require a longer or shorter time, and this will be the subject of subsequent research.

### RESULTS

The clonogenic cell assay was used to measure cell survival after the HX32 tumours were exposed by injecting host mice with each cytotoxic agent. Fig. 1 shows the sensitivity of cells in this tumour, measured 20 h after a single

FIG. 1.—Sensitivity of HX32 tumour cells to drug exposure in mice. Surviving fraction is plotted against drug dose as a fraction of $LD_{10}$. ● = melphalan, □ = HMM, ○ = *cis*-Pt(II), ▼ = thio-TEPA, ▽ = chlorambucil, × = methotrexate, ▲ = vinblastine, △ = adriamycin. S.e. of SF at the $LD_{10}$ dose are shown.

injection of each drug (tumours were left *in situ* for 20 h to allow completion of drug metabolism and to allow for any *in vivo* repair of drug damage). Drug dose has been expressed as a fraction of the $LD_{10}$, which is defined as the single i.p. dose of each agent which kills 10% of mice within 30 days of injection. The $LD_{10}$ data were obtained from the literature and from tests in CBA/lac mice exposed 2–3 weeks after 900 rad immunosuppressive treatment. The assumption has been made that $LD_{10}$ doses, which are by definition equitoxic to mice, are in proportion to the maximum tolerated doses in man. There is a basis for this assumption in reports comparing drug doses in small-animal lethality studies with maximum tolerated doses in human beings (Freireich *et al.*, 1966; Mellett, 1974; Goldsmith *et al.*, 1975).

Table I summarizes the *in vivo* data. Column 2 gives the $LD_{10}$ values used and

TABLE I.—*Ranking of chemotherapeutic agents for cytotoxic effect* in vivo *against clonogenic cells of a human tumour xenograft* (*HX*32)

| Drug | $LD_{10}$ (mg/kg in mice) | Surviving fraction at $LD_{10}$ (from Fig. 1) | *P* | Cytotoxic drug rank *in vivo* |
|---|---|---|---|---|
| Melphalan | 14·0 | 0·004 | | 1 |
| | | | <0·01 | |
| HMM | 350 | 0·009 | | 2 |
| | | | <0·001 | |
| *cis*-Pt(II) | 13·5 | 0·024 | | 3 |
| | | | <0·01 | |
| thio-TEPA | 17·0 | 0·038 | | 4·5 |
| | | | NS | |
| Chlorambucil | 16·5 | 0·044 | | 4·5 |
| | | | <0·001 | |
| Methotrexate | 200 | 0·558 | | 6·5 |
| | | | NS | |
| Vinblastine | 3·0 | 0·670 | | 6·5 |
| | | | <0·05 | |
| Adriamycin | 9·0 | 1·10 | | 8 |

Column 3 gives the surviving fraction of tumour cells at that dose. This fraction was read for each drug at the intersection of the dose–response curve with the vertical dotted line drawn at 1·0 on the abscissa. Fig. 1 shows the standard error of these values, as calculated from the regression analysis used to draw the curves, and the *t* test was used for differences between pairs of drugs. The resulting probabilities are shown in Table I and drugs are ranked accordingly. Column 4 gives the rank of each agent in order of decreasing cytotoxicity as used in the mouse.

### In vitro *cell survival*

Figs. 2, 3 and 4 show the survival of clonogenic tumour cells exposed *in vitro* for 1 h to the 8 cytotoxic drugs. Data points shown are from at least 2 experiments for each drug. The concave form of the curves has been noted in other studies *in vitro* (*e.g.* Barranco *et al.*, 1978). The *in vivo* and *in vitro* dose–response curves for HMM in Figs. 1 and 3 are qualitatively different. The *in vitro* data show a plateau-type response, with little reduction in

FIGS. 2, 3 and 4.—Sensitivity of HX32 tumour cells to drug exposure *in vitro*. Data points from at least 2 experiments are shown. ⇐ indicates survivals at the human plasma concentration over the peak hour (Level A from Table II). ⇐ indicates survival at human plasma concentration over the total time measured (Level B from Table II). (Symbols as in Fig. 1.)

surviving fraction between 1 and 10 μg/ml, whereas the *in vivo* data are fitted by an exponential curve for doses above 0·5× $LD_{10}$. Rutty & Connors (1977) obtained low cytotoxicity of HMM *in vitro* in the absence of liver microsomes, and concluded that this drug owes its cytotoxicity

FIG. 2.—Sensitivity of HX32 tumour cells to chlorambucil, *cis*-Pt(II) and vinblastine *in vitro*. (Symbols as in Fig. 1.)

FIG. 4.—Sensitivity of HX32 tumour cells to melphalan and adriamycin *in vitro*. (Symbols as in Fig. 1.)

FIG. 3.—Sensitivity of HX32 tumour cells to thio-TEPA, HMM and methotrexate *in vitro*. (Symbols as in Fig. 1.)

FIG. 5.—A comparison of the *in vitro* action of HMM with that of its postulated active metabolite, pentamethyl monomethylol melamine. Data from 3 experiments.

to activation *in vivo*. Our method of *in vitro* exposure would, therefore, underestimate the possible effect of HMM in patients at concentrations above 1 μg/ml. We have therefore excluded HMM from the *in vitro*/*in vivo* comparison. A sample of the postulated active metabolite of HMM (pentamethyl monomethylol melamine) was kindly given to us by C. J. Rutty. This compound showed no plateau of response (Fig. 5). These results therefore support the conclusion that activation of HMM is needed for maximum cytotoxic effect.

In this preliminary study we have assessed the *in vitro* sensitivity of cells from HX32 tumour at concentrations derived by 2 alternative methods from human plasma clearance curves:

(a) On the hypothesis that the initial peak levels of drug determine the response, the average concentration was measured over the first hour after administration (or the average between 1·5 and 2·5 h after oral HMM) and these values are quoted as Level A in Table II. The solid arrows in Figs. 2–4 show the fraction of cells which survived incubation with Level A for 1 h.

(b) On the hypothesis that drug concentration × time is the effective parameter of drug exposure, the integral of the whole curve was measured. We then estimated the effect of exposing cells for 1 h at the concentration (Level B) that would give the same integral dose (concentration × time). The open arrows in Figs. 2–4 indicate the fraction of cells which survive 1 h incubation *in vitro* at Level B. The surviving fractions at the positions of these arrows were used to rank drugs for *in vitro* activity, as shown in Table III. For HMM, Levels A and B are both much higher than the concentration at which the plateau level of cell kill was reached *in vitro* and this drug therefore was omitted from the *in vitro* ranking.

*Comparison of* in vivo *and* in vitro *assessments of chemosensitivity*

As shown in Table III, there is good correlation between *in vitro* ranking based on plasma concentrations and *in vivo* ranking. The ranking at 1h plasma levels correlates more closely to ranking at mouse $LD_{10}$ doses than does the ranking at the doses equivalent to the total plasma clearance curve (Spearman rank correla-

TABLE II.—*Integrals on drug concentration in human plasma* (*μg/ml/h*)

| Drug | Dose per patient (mg) | A* | B† | Reference |
|---|---|---|---|---|
| Melphalan | 30 | 0·77 | 1·28 (3) | Tattersall *et al.*, 1978 |
| HMM | 12/kg | 14 | 114 (24) | Bryan *et al.*, 1968 |
| *cis*-Pt (II) | 20/m² | 1·92 | 20·2 (20) | Malerbi (pers. comm.) |
| thio-TEPA | 0·3/kg | 0·19 | 0·35 (4) | Mellett *et al.*, 1962 |
| Chlorambucil | 10 | 0·18 | 0·51 (6) | D. Newell (pers. comm.) |
| Methotrexate | 200 | 1·07 | 3·18 (20) | Calvert *et al.*, 1978 |
| Vinblastine | 12–16 | 0·136 | 0·32 (4) | Owellen *et al.*, 1977 |
| Adriamycin | 60/m² | 0·3 | 1·65 (48) | Benjamin *et al.*, 1977 |

* Integral over the period 0–1 h after administration (1·5–2·5 h for HMM).
† Integral of the whole plasma clearance curve up to the time (in h) shown in brackets.

TABLE III.—In vitro *cytotoxicity of* 8 *drugs against HX*32 *cells*

| Drug | Surviving fraction at level A* (solid arrows) | Rank (A) | Surviving fraction at level B* (open arrows) | Rank (B) | *In vivo* rank† |
|---|---|---|---|---|---|
| Melphalan | 0·17 | 1 | 0·041 | 2 | 1 |
| *cis*-Pt (II) | 0·29 | 2 | 0·012 | 1 | 2 |
| Thio-TEPA | 0·35 | 3 | 0·29 | 4 | 3·5 |
| Chlorambucil | 0·42 | 4 | 0·16 | 3 | 3·5 |
| Adriamycin | 0·88 | 5 | 0·54 | 5 | 7 |
| Methotrexate | 0·93 | 6 | 0·88 | 6 | 5·5 |
| Vinblastine | 1·0 | 7 | 1·0 | 7 | 5·5 |

* From Table II.

† From Table I, omitting HMM.

tion coefficients are 0·873 and 0·836 respectively). This difference is due to the reversal of the ranks of melphalan and *cis*-Pt(II). The long-term *cis*-Pt(II) estimate may be too high, as the Pt ion itself was measured (using the method of Malerbi & Abel, 1977) in the plasma up to 20 h after injection, whereas intact molecules of *cis*-Pt may no longer be present. The major discrepancy in ranking was adriamycin (ranked 5 *in vitro* but killing no cells *in vivo*).

### DISCUSSION

The good correlation that we have found between *in vitro* and *in vivo* effects of drugs lends support to our original hypothesis. We had proposed that predictions of the relative efficacy of drugs against tumour cells in patients might be made:

(a) by assessing cytotoxicity against human tumour cells grown in immune-suppressed mice at doses of drug equitoxic to mice, and
(b) by assessing cytotoxicity to human tumour cells *in vitro* at drug concentrations found in patients.

Although at the present time there are unavoidable uncertainties in the translation of drug levels from *in vivo* to *in vitro*, and from man to mouse, this study has shown that plausible assumptions lead to a good correlation between responses seen *in vitro* and in the mouse. However, it was found to be impossible to assess HMM by an *in vitro* method because of its requirements for *in vivo* activation, and adriamycin, a drug with a wide spectrum of clinical activity, failed to show *in vivo* cell kill to the extent that would have been predicted by our *in vitro* studies. Other investigators have found adriamycin to be ineffective against mouse tumours, and this has been attributed to poor drug access (Sutherland *et al.*, 1979).

The validity of the rankings obtained in this study depends on the errors involved in assessing cell survival at a given drug dose. For HX32 cells the *in vivo* dose–response curves for the 8 agents are exponential in most cases, and values for surviving fraction (SF) at the $LD_{10}$ and its standard error can be calculated from the regression analysis. This enables the significance of differences between cytotoxicity of different drugs at the $LD_{10}$ to be determined (assuming an accurate $LD_{10}$ estimate). The pooling of data from several mouse strains and several laboratories should give a good estimate of $LD_{10}$.

The *in vitro* dose–response curves are less well defined than the *in vivo* curves, but they are generally not exponential and have been drawn by eye. Thus the errors of SF for a given dose are not known unless experimental observations have by chance been made at that desired concentration. However, Table III shows that our assessment of drugs *in vitro* produces similar drug rankings, whether the effect is measured at the peak human plasma concentration or at the drug level equivalent to the integral of the whole plasma clearance curve. Furthermore, both *in vitro* rankings correlate well with drug ranks assessed *in vivo*.

Salmon *et al.* (1978), using an alternative approach for defining *in vitro* sensitivity of cells from tumour biopsies, compared *in vitro* results with the response of patients. Their distinction between "resistance" and "sensitivity" *in vitro* was somewhat arbitrary, being based on the integral of a cell-survival *vs* drug-concentration curve with upper limits of 0·1 μg/ml for melphalan and bleomycin, and 0·2 μg/ml for the other drugs used. Available pharmacological data indicate that melphalan, for example, gives a peak plasma level in patients of ~1 μg/ml (Tattersall *et al.*, 1978), which is higher than the maximum concentration used *in vitro* by Salmon and his colleagues (1978). If the 8 drugs of the present study had been assessed *in vitro* at a concentration of 1 μg/ml, chlorambucil would have been ranked as the most effective drug followed by (2) melphalan, (3) thio-TEPA, (4) *cis*-Pt(II), (5) adriamycin, (6) methotrexate and (7) vinblastine. This correlates poorly with the *in vivo* ranking ($P > 0{\cdot}05$ for a Spearman rank correlation of 0·67). Thus the use of arbitrary drug concentrations *in vitro* precludes any effective ranking of drugs.

For the 8 drugs in this study, we conclude that HMM and adriamycin cannot be used *in vitro* to mimic *in vivo* response. For the other 6 drugs, 1h *in vitro* exposures can be used to predict the *in vivo* effect of a single injection, if *in vitro* concentrations approximating to drug levels in patients' plasma are used.

Theoretically, the measurement of drug cytotoxicity at human plasma concentrations *in vitro* and at doses equitoxic to mice *in vivo* might both be expected to correlate with cytotoxicity in patients at drug doses equitoxic to man. The use of both these methods must be validated by studies on biopsy material from many human cancers and correlation of laboratory results with patients' responses. However, we have demonstrated, using one human tumour xenograft, that the 2 assays correlate well with one another, and we therefore feel encouraged in our attempts to use the *in vitro* test for alkylating agents and *cis*-Pt(II) to compare the response of tumour biopsy material with patient response to chemotherapy. A study of this type is in progress, using ovarian carcinoma cells.

The authors acknowledge the able assistance of Graham Towse and are indebted to Doreen Courtenay for the development of the clonogenic assay used in this study. We are grateful to D. Newell of this Institute and B. W. Malerbi of the Johnson Matthey Research Centre for their data on plasma levels of chlorambucil and *cis*-Pt(II) respectively.

The study was funded by the National Cancer Institute Grant No. RO1 CA 20519.

### REFERENCES

Barranco, S. C., Haenelt, B. R. & Gee, E. L. (1978) Differential sensitivities of five rat hepatoma cell lines to anticancer drugs. *Cancer Res.*, **38,** 656.

Benjamin, R. S., Riggs, C. E., Jr & Bachur, N. R. (1977) Plasma pharmokinetics of adriamycin and its metabolites in humans with normal hepatic and renal function. *Cancer Res.*, **37,** 1416.

Berry, R. J., Laing, A. H. & Wells, J. (1975) Fresh explant culture of human tumours *in vitro* and the assessment of sensitivity to cytotoxic chemotherapy. *Br. J. Cancer*, **31,** 218.

Bryan, G. T., Worzalla, J. F., Gorske, A. L. & Ramirez, G. (1968) Plasma levels and urinary excretion of hexamethylmelamine following oral administration to human subjects with cancer. *Clin. Pharmacol. Ther.*, **9,** 777.

Calvert, A. H., Bondy, P. K. & Harrap, K. R. (1978) Some observations on the human pharmacology of methotrexate. *Cancer Treatment Rep.*, **61,** 1647.

Courtenay, V. D. & Mills, J. (1978) An *in vitro* colony assay for human tumours grown in immune-suppressed mice and treated *in vivo* with cytotoxic agents. *Br. J. Cancer*, **37,** 261.

Freireich, E. J., Gehan, E. A., Rall, D. P., Schmidt, L. H. & Skipper, H. E. (1966) Quantitative comparison of toxicity of anticancer agents in mouse, rat, hamster, dog, monkey and man. *Cancer Chemother. Rep.*, **50,** 219.

Goldsmith, M. A., Slavik, M. & Carter, S. K. (1975) Quantitative prediction of drug toxicity in humans from toxicology in small and large animals. *Cancer Res.*, **35,** 1354.

Houghton, P. J., Houghton, J. A. & Taylor, D. M. (1977) Effects of cytotoxic agents on TdR incorporation and growth delay in human colonic tumour xenografts. *Br. J. Cancer*, **36,** 206.

Malerbi, B. W. & Abel, G. (1977) Pharmacokinetics of platinum antitumour agents in mouse organs. (Abstr.) *Br. J. Cancer*, **36,** 420.

MELLETT, L. B. (1974) Pharmacodynamic and pharmacokinetic measurements of antitumour agents. *Clin. Pharmacol. Ther.*, **16**, 230.

MELLETT, L. B., HODGSON, P. E. & WOODS, L. A. (1962) Absorption and fate of $C^{14}$-labelled N,N',N''-triethylenethiophosphoramide (thio-Tepa) in humans and dogs. *J. Lab. Clin. Med.*, **60**, 818.

MITCHELL, J. S., DENDY, P. P., DAWSON, A. M. P. & WHEELER, T. K. (1972) Testing anticancer drugs. *Lancet*, i, 955.

NOWAK, K., PECKHAM, M. J. & STEEL, G. G. (1978) Variation in response of xenografts of colo-rectal carcinoma to chemotherapy. *Br. J. Cancer*, **37**, 576.

OWELLEN, R. J., HARTKE, C. A. & HAINS, F. O. (1977) Pharmacokinetics and metabolism of vinblastine in humans. *Cancer Res.*, **37**, 2597.

RUTTY, C. J. & CONNORS, T. A. (1977) *In vitro* studies with hexamethylmelamine. *Biochem. Pharmacol.*, **26**, 2385.

SALMON, S. E., HAMBURGER, A. W., SOEHNLEN, B., DURIE, B. G. M., ALBERTS, D. S. & MOON, T. E. (1978) Quantitation of differential sensitivity of human tumour cells to anticancer drugs. *N. Engl. J. Med.*, **298**, 1321.

STEEL, G. G., COURTENAY, V. D. & ROSTOM, A. Y. (1978) Improved immune-suppression techniques for the xenografting of human tumours. *Br. J. Cancer*, **37**, 224.

SUTHERLAND, R., BAREHAM, B., VAN ANTWERP, D. & REICH, K. (1979) Analysis of factors influencing responses to adriamycin using the multicellular spheroid tumour model. *Int. J. Radiat. Oncol., Biol. Phys.* (in press).

TATTERSALL, M. H. N., JARMAN, M., NEWLANDS, E. S., HOLYHEAD, L., MILSTED, R. A. V. & WEINBERG, A. (1978) Pharmaco-kinetics of melphalan following oral or intravenous administration in patients with malignant disease. *Eur. J. Cancer*, **14**, 507.